# FREE-LIVING HEART RATE VARIABILITY/PHYSICAL ACTIVITY AND COGNITIVE FUNCTION AND DEMENTIA IN ARIC

**DOI:** 10.1093/geroni/igad104.1169

**Published:** 2023-12-21

**Authors:** Francesca Marino, Hau-Tieng Wu, Mary Rooney, Elsayed Soliman, Jennifer Deal, Amal Wanigatunga, Jennifer Schrack, Lin Yee Chen

**Affiliations:** Johns Hopkins Bloomberg School of Public Health, Baltimore, Maryland, United States; Duke University, Durham, North Carolina, United States; Johns Hopkins University Bloomberg School of Public Health, Baltimore, Maryland, United States; Wake Forest University School of medicine, Winston-Salem, North Carolina, United States; Johns Hopkins University, Baltimore, Maryland, United States; Johns Hopkins School of Public Health, Baltimore, Maryland, United States; Johns Hopkins University, Baltimore, Maryland, United States; University of Minnesota School of Medicine, Minneapolis, Minnesota, United States

## Abstract

Low heart rate variability (HRV) and physical activity (PA) are associated with cognitive dysfunction in clinical settings. Yet, associations with these measures among older adults in the free-living environment remain unexplored. We hypothesize higher free-living HRV and PA are associated with higher cognitive test scores and less likelihood of mild cognitive impairment (MCI)/dementia. This study includes 1,590 ARIC participants aged 72-94 years who wore the Zio XT Patch for 14-days to simultaneously capture ECG and accelerometry, from which we derived HRV as the standard deviation (SDNN) or root mean squared successive difference (rMSSD) of normal RR intervals and PA as total mean amplitude deviation (TMAD; higher values represent more movement). Linear or ordinal regression models were fitted to estimate cross-sectional associations between log transformed HRV or PA with latent-variable derived global and domain-specific cognitive factor scores or adjudicated MCI/dementia, respectively. Participants were on average 79 years, 57% female, and 32% Black. There were no significant associations between either HRV measure and cognition. After adjusting for demographic and medical covariates, 1-unit higher in log TMAD was associated with a 0.30-point higher global factor score (95% CI: 0.16-0.44), 0.38-point higher executive function factor score (95% CI: 0.22-0.53), 62% lower odds of MCI (95% CI: 0.22-0.67) and 75% lower odds of dementia compared to normal cognition (95% CI: 0.08-0.74). In summary, free-living PA, but not HRV of RR intervals, is associated with cognitive and dementia status. Further investigation of other objective HRV measures and directionality of associations between PA and cognition is needed.

